# Supplementary material for: Learning curve and surgical outcome of robotic assisted colorectal surgery with ERAS program
Source: Sci Rep. 2022 Nov 29;12:20566. doi: 10.1038/s41598-022-24665-w (PMC9709162; doi:10.1038/s41598-022-24665-w)
Supplement: Supplementary file 2 — Supplementary Information 2. [file 41598_2022_24665_MOESM2_ESM.zip › RAL Raw data-Ñ[▒K/076/OP_ñw╝╨░O▒Kñσ.pdf]

正式摘要(電子簽章：000000215575858)

請選擇...reporter

臺中榮民總醫院

Operation Note

[redacted]

- 手術日期：2021/04/21
- 手術醫師：VS 林俊余，CR 林陳彥，R 陳威遠
- Anesthesia：GE
- Position：Lithotomy
- Aseptic agents：Betadine Aqua，Zephnan

Diagnosis

- Pre-operative diagnosis : Malignant tumor of sigmoid colon
- Post-operative diagnosis : s/p robotic anterior resection

Surgical Indication

- For removing the primary tumor

Operation

- 1. Robotic (XI) assisted anterior resection
  2. Intraoperative colonoscopy : (-).
  3. High ligation (+) : At IMA root. Left colic artery and IMV were also ligated via robotic endoclips.
  4. Take down from the splenic flexure : (-)
  5. Robotic endoGIA (45-4.3) X2 + EEA (29mm) were applied
  6. Proximal cut end: 7 cm, distal cut end: 2 cm
  7. Hyalobarrier gel was placed below the LLQ abdominal wound

Finding

- 1. A 5 x 5 cm, ulcerative tumor at S-colon.
  2. No obvious ascites
  3. No obvious visible lesions were found over the liver surface
  4. Bilateral doughnuts : intact ; Air leak test: Done and no air leak was found
  5. ICG test : (Post resection via robotic endoGIA) About 30 secs

Procedure

- 1. General endotracheal anesthesia was induced and then the patient was placed in modified Trendelenburg lithotomy position.
  2. Preoperative antibiotics were given. A Foley catheter was placed.
  3. The abdomen was prepped and draped in standard sterile fashion.
  4. A transverse incision was made above the umbilicus and a 8 mm trocar was inserted into the peritoneal cavity by open method. Peumoperitoneum was established with CO2 inflation.
  5. Laparoscopy was inserted and then another 8mm trocar was inserted over LUQ area of abdomen. A 12 mm trochar over RLQ area of abdomen. Another 8mm trochar over RLQ of abdomen. Finally, a 12 mm trocar was inserted over RUQ area as an assist-port. The operative findings were mentioned as above.
  6. The sigmoid colon was held by grasper and the peritoneum overlying the mesentery was scored with electrocautery.
  7. The inferior mesentery artery was identified, ligated above the left colic artery with robotic endoclips, and transected.
  8. Dissection was done above the Gerota’s fascia and toward the peritoneal attachments.
  9. The colon was mobilized from retroperitoneum overlying the sacral promontory and into the pelvis.

- 10. Peritoneal reflection was not resected.
  11. Distal descending colon and s-colon were freely mobilized after the mesoclon was all dissected.
  12. The distal s-colon was divided with Robotic endoGIA (45-4.3) X2.
  13. A new wound was made over the LLQ of abdomen. We approached into the peritoneal cavity by open method. S-colon was pulled out.
  14. The proximal colon was then taken out for carefully inspection to make sure it has an excellent blood supply. ICG test was performed. The result was about 30 secs.
  15. The field was isolated with moist pad and the bowel opened and sized. It was determined that a 29 mm circular stapler could be accommodated. A purse string suture of 2-0 prolene was placed. The tumor-containing tissue was removed. Then the anvil was inserted, followed by the purse string suture. The colon was then put back to the abdominal cavity.
  16. Pneumoperitoneum was constructed again.
  17. The anal canal was irrigated with distil water and then the circular stapler was introduced through the anus and pierced through the upper area (The ventral site of the body) of the stapler line.
  18. The proximal bowel with anvil in place was engaged with the circular stapler and the stapler was then fired. Two complete donuts were obtained and the distal one was submitted to pathology for margin confirmation.
  19. The anastomosis was tested for leaks with irrigation solution and air introduced through the rectum and was found to be patent and intact.
  20. The trochar fascia wounds were all closed with interrupted sutures via 1-O Vicryl.
  21. Hyalobarrier gel was placed below the LLQ abdominal wound.
  22. The LLQ fascia wound was closed with interrupted sutures via Num.1 Vicryl.
  23. Massive N/S was applied to irrigate the wound.
  24. The wound was further closed with 3-O Nylon, then covered with Medipore.
  25. The patient tolerated the procedure well and was brought to the POR in stable condition.
- Estimated blood loss: minimum
- 器械數量: 正確
- 紗布數量: 正確

Image

- PAINT
  - abdomen

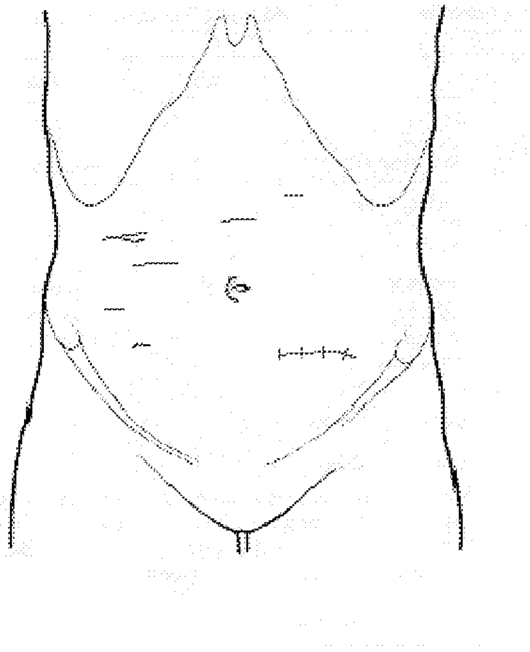

  - colon & rectum

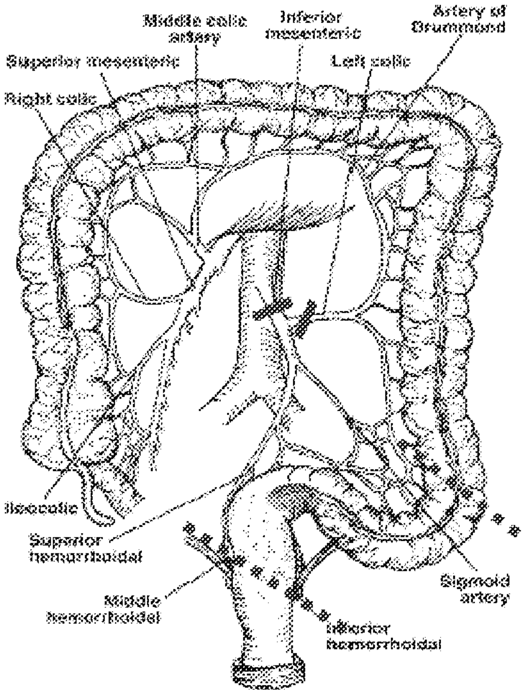

- The resected S-colon

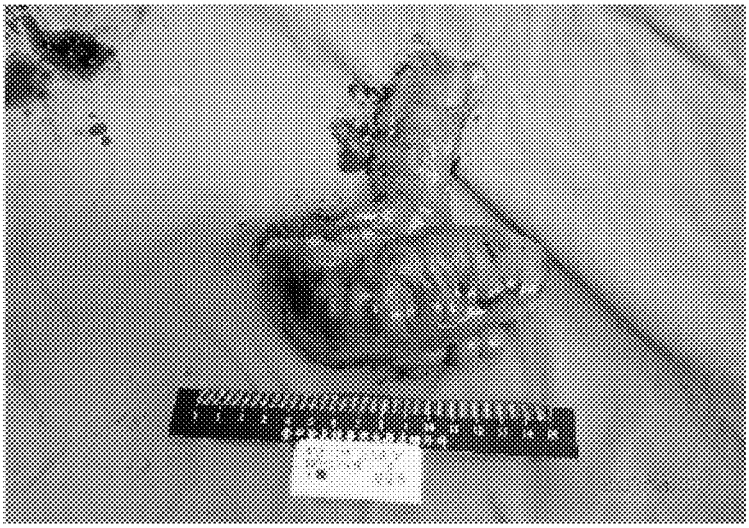

The tumor could be clearly seen

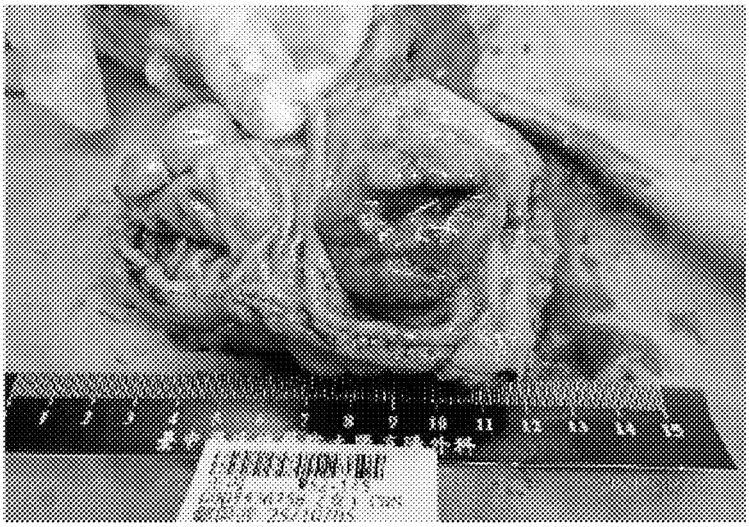

主治 2222H林俊余 / 醫師 2301H林陳彥

正式摘要(電子簽章：**000000215575858)**

請選擇...reporter

doctorRank:B
